# California State Dental Law

**Published:** 1885-05

**Authors:** 


### Editorial, Etc.

California State Dental Law.—An Act to insure the better education of practitioners of dental surgery, and to regulate the practice of dentistry in the State of California, approved March 12, 1885.

*The People of the State of California, represented in Senate and Assembly, do enact as follows:*

Section 1. It shall be unlawful for any person who is not at the time of the passage of this Act engaged in the practice of dentistry in this State to commence such practice, unless he or she shall have obtained a certificate as hereinafter provided.

Sec. 2. A Board of Examiners, to consist of seven practicing dentists, is hereby created, whose duty it shall be to carry out the purposes and enforce the provisions of this Act. The members of said Board shall be appointed by the Governor from the dental profession of the State at large. The term for which the members of said Board shall hold their offices shall be four years, except that two of the members of the Board first to be appointed under this Act, shall hold their office for the term of one year, two for the term of two years, two for the term of three years, and one for the term of four years, respectively, and until their successors shall be duly appointed and qualified. In case of a vacancy occurring in said Board, such vacancy shall be filled by the Governor in conformity with this section.

Sec. 3. Said Board shall choose one of its members President, and one the Secretary thereof, and it shall meet at least once in each year, and as much oftener and at such times and places as it may deem necessary. A majority of said Board shall, at all times, constitute a quorum, and the proceedings thereof shall, at all reasonable times, be open to public inspection.

SEC. 4. Within six months from the time that this Act takes affect, it shall be the duty of every person who is now engaged in the practice of dentistry in this State to cause his or her name and residence or place of business to be registered with said Board of Examiners, who shall keep a book for that purpose. The statement of every such person shall be verified under oath before a Notary Public or Justice of the Peace in such a manner as may be prescribed by the Board of Examiners. Every person who shall so register with said Board as a practitioner of dentistry shall receive a certificate to that effect, and may continue to practice as such without incurring any of the liabilities or penalties provided in this Act, and shall pay to the Board of Examiners for such registration a fee of one dollar. It shall be the duty of the Board of Examiners to forward to the County Clerk of each county in the State, a certified list of the names of all persons residing in his county who have registered in accordance with the provisions of this Act, and it shall be the duty of all County Clerks to register such names in a book, to be kept for that purpose.

SEC. 5. Any and all persons, who shall so desire, may appear before said Board at any of its regular meetings and be examined with reference to their knowledge and skill in dental surgery, and if the examination of any such person or persons shall prove satisfactory to said Board, the Board of Examiners shall issue to such persons as they shall find to possess the requisite qualifications a certificate to that effect, in accordance with the provisions of this Act. Said Board shall also indorse as satisfactory diplomas from any reputable dental college, when satisfied of the character of such institution, upon the holder furnishing evidence satisfactory to the Board of his or her right to the same, and shall issue certificates to that effect within ten days thereafter. All certificates issued by said Board shall be signed by its officers, and such certificates shall be prima facie evidence of the right of the holder to practice dentistry in the State of California.

SEC. 6. Any person who shall violate any of the provisions of this Act, shall be deemed guilty of a misdemeanor, and, upon conviction, may be fined not less than fifty doliars nor more than two hundred dollars, or confined six months in the county jail for each and every offense. All fines recovered under this Act shall be paid into the Common School Fund of the county in which such conviction takes place.

SEC. 7. In order to provide the means for carrying out and maintaining the provisions of this Act, the said Board of Examiners shall charge each person applying to or appearing before them for examination for a certificate of qualifications, a fee of ten dollars, which fee shall in no case be returned, and out of the funds coming into the possession of the Board from the fees so charged, and penalties received under the provisions of this Act, all legitimate and necessary expenses incurred in attending the meetings of said Board shall be paid. And no part of the expenses of the Board shall ever be paid out of the State Treasury. All moneys received in excess of expense, above provided for, shall be held by the Secretary of said Board as a special fund for meeting the expenses of said Board, and carrying out the provisions of this Act, he giving such bonds as the Board shall from time to time direct. And said Board shall make an annual report of its proceedings to the Governor, by the first of December of each year, together with an account of all moneys received and disbursed by them pursuant to this Act.

SEC. 8. Any person who shall receive a certificate from said Board to practice dentistry, shall cause his or her certificate to be registered with the County Clerk of the county in which such person may reside, and the County Clerk shall charge for registering such certificate a fee of one dollar. Any failure, neglect, or refusal on the part of any person holding such certificate to register the same with the County Clerk as above directed, for a period of six months, shall work a forfeiture of the certificate, and no certificate, when once forfeited shall be restored, except upon the payment to the said Board of Examiners of the sum of twenty-five dollars, as a penalty for such neglect, failure, or refusal.

SEC. 9. Any person who shall knowingly and falsely claim or pretend to have or hold a certificate of license, diploma, or degree, granted by any society organized under and pursuant to the provisions of this Act, or who shall falsely and with intent to deceive the public, claim or pretend to be a graduate from any incorporated dental college, shall be deemed guilty of a misdemeanor, and shall be liable to the same penalty as provided in section six.

SEC. 10. Nothing in this Act shall be so construed as to prohibit any practicing physician from extracting teeth.

SEC. 11. This Act shall take effect immediately.

The members of the Board appointed by the Governor as follows:

| | |
|---|---|
| S. W. Dennis, M. D., D. D. S., *President*, - | San Francisco. |
| E. W. Biddle, - - - - - | Healdsburg. |
| J. W. Hollingsworth, - - - | Los Angelos. |
| Thos. Morffew, D. D. S., - - | San Francisco. |
| S. S. Southworth. - - - - | Sacramento. |
| M. J. Sullivan, D. D. S. - - - | San Francisco. |
| Chas. W. Hilbard, D. D. S., *Secretary*, | San Francisco. |

Respectfully,
C. W. Hilbard, *Secretary*.

